# Reply to: “Reconsidering low HDL-cholesterol levels as a predictive factor for the development of hepatocellular carcinoma”

**DOI:** 10.1016/j.jhepr.2023.100783

**Published:** 2023-04-28

**Authors:** Lucilla Crudele, Carlo De Matteis, Antonio Moschetta

**Affiliations:** 1 Department of Interdisciplinary Medicine, University of Bari “Aldo Moro”, 70124 Bari, Italy

To the Editor:

We are grateful to Dr. Fujii and colleagues^1^ for their interest in our study^2^ and for taking the time to validate our findings and investigate the predictive role of HDL cholesterol (HDL-C) in the progression from liver fibrosis to hepatocellular carcinoma (HCC). We welcome this opportunity to discuss the rationale for assessing lipid profile and liver fibrosis as a prevention strategy for HCC.

In their letter, the authors suggest that the use of HDL-C level in HCC prediction may be premature, since in their population of Japanese patients with biopsy-proven NAFLD, HCC occurrence did not differ between groups of patients depending on HDL-C levels. Firstly, we believe that the identification of sub-populations in the study from Fujii *et al.* is quite different from ours. Indeed, from a methodological point of view, while they stratified the population according to HDL-C levels under or above 40 mg/dl, we first subdivided our sample according to liver fibrosis using APRI score ≥0.5, then we analysed the sub-population of patients with HDL-C ≤50 mg/dl to predict HCC, as shown in Fig. 5 of the original paper.^2^ Nonetheless, in line with our findings, Fujii *et al.*^1^ found that HDL-C levels were associated with advanced liver fibrosis, which is the fruitful crib for HCC.

Secondly, our results pointed not only to HDL-C levels, but also to waist circumference and fasting glucose levels (see Fig. 1A,C of the original study^2^) as anthropometric and bio humoral predictive biomarkers. Indeed, an important contributor to the overall pool of HDL-C could be adipose tissue, since adipocytes in culture have the ability to efflux cholesterol to HDL particles.^3^ More specifically, the amount of visceral adipose tissue is related to increased systemic inflammation and cancer risk and may represent a reservoir of HDL.^4^ Thus, anthropometric and clinical features should be considered when characterizing the population and, at variance to data from Fujii *et al.*, in our study visceral adiposity and fasting glycemia play a role together with HDL-C in predicting risk of HCC in patients with liver fibrosis.

On a different angle, we would lift the veil also on HDL function rather than HDL-C levels *per se*. Indeed, some studies have called into question the hypothesis that the static measurement of HDL-C levels may not perfectly reflect the functional effects of HDL *in vivo* in genetically different populations. For instance, HDL-C levels do not correlate with the cholesterol efflux capacity of macrophages, a measure of cholesterol efflux from peripheral tissues to the liver.^5^ Unidirectional flux of cholesterol from macrophages to lipid-free or lipid-poor apolipoproteins, which represents the first step of reverse cholesterol transport, is promoted by transporters belonging to the ATP binding cassette superfamily, namely ABCA1 and ABCG1.^6^ Both are transcriptionally regulated by nuclear liver X receptor (LXR)-α/β, which are activated by cholesterol-derived oxysterols. In normal conditions, when the intracellular levels of oxysterols increase, LXR upregulates cholesterol efflux, thus elegantly maintaining its homeostasis. Conversely, when hepatocyte proliferation is required, turning off LXR-transcriptional pathways is crucial to guaranteeing the intracellular cholesterol pool needed for cellular growth.^7^ Similarly, in cancer cells, despite the intracellular cholesterol abundance, LXR is downregulated due to the metabolic shift of oxysterol metabolism from anabolic toward catabolic pathways.^8^ Thus, from a mechanistic point of view, the reduction of reverse cholesterol transport could be related to increased cancer risk even more than the reduction of HDL-C level*s*. Moreover, HDL level and function differ between Japanese and Western populations depending on nutritional habits, obesity and metabolic syndrome criteria^9^ and on modifications of HDL proteins and lipids that affect HDL functionality.^10^

In conclusion, we agree on the need for further studies to validate the use of HDL in HCC prediction, but evidence about the association of HDL-C and liver fibrosis that also come from Fujii *et al.’*s study confirm our hypothesis on the pathogenic involvement of HDL dysfunction in liver fibrosis and cancer development, at least in individuals with visceral adiposity.

## Financial support

A.M. is funded by Italian Association for Cancer Research - AIRC IG 2019 Id 23239.

## Authors’ contributions

Writing—original draft preparation, L.C. and C.D.M.; writing—review and editing, A.M. All authors have read and agreed to the published version of the manuscript.

## Conflict of interest

The authors declare no conflict of interest.

Please refer to the accompanying ICMJE disclosure forms for further details.
